# Concept Development and Field Testing of Wireless Outdoor Indicator System for Use in Monitoring Exposures at Work among Malaysian Traffic Police

**DOI:** 10.3390/toxics11040385

**Published:** 2023-04-18

**Authors:** Putri Anis Syahira Mohamad Jamil, Nur Athirah Diyana Mohammad Yusof, Karmegam Karuppiah, Irniza Rasdi, Vivien How, Shamsul Bahri Mohd Tamrin, Muhammad Hasnolhadi Samsudin, Sivasankar Sambasivam, Nayef Shabbab Almutairi

**Affiliations:** 1 Department of Environmental Engineering, Faculty of Engineering and Green Technology, Universiti Tunku Abdul Rahman, Jalan Universiti, Bandar Barat, Kampar 31900, Malaysia; 2 Engineering and Technology Department, Razak Faculty of Technology and Informatics, Universiti Teknologi Malaysia Kuala Lumpur, Kuala Lumpur 54100, Malaysia; 3 Department of Environmental and Occupational Health, Faculty of Medicine and Health Sciences, Universiti Putra Malaysia, Serdang 43400, Malaysia; 4 Department of Construction Management, Faculty of Engineering and Technology, Universiti Tunku Abdul Rahman, Kampar 31900, Malaysia; 5 Department Public Health, Al-Lith College of Health Sciences, Umm Al-Qura University, P.O. Box 3712, Mecca 21955, Saudi Arabia

**Keywords:** ambient air monitoring, air pollution, wireless sensor, occupational exposure, field evaluation

## Abstract

Real-time exposure air monitoring is essential to protect the respiratory health of the Malaysian traffic police. However, the data from monitoring stations have been inadequate to provide accurate information about their exposure. This report describes the conceptual design of a wireless exposure indicator system, and then evaluates the field performance of the system by collocation. The study tested the accuracy of particulate matter size 2.5 (PM_2.5_), carbon monoxide (CO), and nitrogen dioxide (NO_2_) by comparing the measurements from the prototype with the measurements from reference instruments. The field testing found that the data tested were significantly correlated with each other (PM_2.5_-rs = 0.207, *p* = 0.019; NO_2_-rs = 0.576, *p* = 0.02 and CO-rs = 0.545, *p* = 0.04). The prototype proved to be successful as it can compute and transmit real-time monitoring data on the level of exposure to harmful air.

## 1. Introduction

Malaysia is facing a drawback in safety and health due to rapid industrialization and urbanization, with increased pollution (notably air pollution) resulting in a lower quality of life and reduced life expectancy [1]. Referring to an article by the World Health Organization (WHO) (2018), air pollution will leave an impression on those who were exposed to it. Polluted air can cause respiratory irritation or breathing difficulties even for healthy people [2]. The actual risk depends on the current health status, the pollutant type and concentration, and the length of exposure to polluted air [3]. Despite that, traffic police, especially, are directly exposed to polluted air daily as they constantly work outdoors. This is in line with several studies which found that these traffic police are affected in terms of their respiratory health due to the exposure to high concentrations of particulate matter in the polluted air [4,5]. With N95 face masks as their only protection in congested areas daily for eight working hours, it is simply not enough to protect their respiratory health [4]. 

Air pollution monitoring is used for many purposes, from improving the quality of life to military operations, as demonstrated by El Raey [6]. However, conventional methods are not mobile, available only in fixed locations, expensive, have a limited spatial resolution, and are inefficient in communicating the results [7]. Most importantly, the methods to communicate air quality data via government websites are ineffective and troublesome because of the low spatial and temporal resolutions [8]. Although the low spatio-temporal resolution is ideal for environmental monitoring, it is highly inadequate for communities, especially outdoor workers, to know their exposure to air pollution, and cannot represent individual health risks [9]. Maraiya, Kant, and Gupta have demonstrated a new technology equipped with low-cost sensors that can directly track air pollutants and deliver online and real-time results using the Wireless Sensor Network (WSN) [10]. Per the recommendations of the US Environmental Protection Agency (EPA) and the European Union (EU) Directive, various studies have been conducted on the use of real-time air monitoring using low-cost sensors [11,12,13]. The new technology has leverage in terms of low-cost requirements, being mobile, relatively small in size, having low power usage, large coverage area, and most significantly, online availability of data on websites and smartphone applications [7]. Hence, this present study describes an available air monitoring method for ambient air and low-cost sensors used as a new technology, which has attracted attention in recent years. The search concludes on the gap which is the lack of a mobile air monitoring system that provides real-time data communicated wirelessly among the users using low-cost sensors. Reliable data generated from the sensors are also a gap to address. The development of the prototype is explained in the study, focusing on the methodology of its development and initial testing in field deployment.

The necessity for a useful tool that can efficiently assist in the detection of air contaminants was highlighted by several earlier studies conducted among the Malaysian traffic police [14,15]. Implementing an indicator system with real-time exposure to air pollutants allows the traffic police to be alert and aware of the changes in their surrounding environment. This will encourage them to act accordingly when the exposure exceeds the acceptable limit set by the Malaysian Department of Environment (DOE) at any given time. Hence, it is crucial to develop a device that can help monitor and control the level of exposure to outdoor air pollution. Nevertheless, air quality monitoring devices that are currently equipped with this technology are still limited in Malaysia. In response to this problem, this study proposes a conceptual design of a wireless outdoor individual exposure indicator system prototype for the traffic police according to their needs while on duty and conducts field testing for the functionality of each component of the prototype.

## 2. Materials and Methods

### 2.1. The System

One promising technology for monitoring ambient air pollution is using low-cost sensors, which have mobile characteristics and low requirements for maintenance [16]. Subsequently, these sensors theoretically allow air pollution monitoring to become feasible in far more locations [17]. In the field of air monitoring, low-cost sensors have a wide range of applications but are not restricted to personal exposure and health monitoring, community monitoring, and ambient air monitoring [18].

These small and lightweight sensors offer wearable applications, an opportunity that could play a key role in determining the effect of air pollution on human health in the future [19]. In addition, the sensors can be incorporated on various mobile and stationary monitoring frameworks to proactively manage pollutant sources and originating regions. With promising results, these sensors have already been evaluated against reference methods [19,20]. 

A real time outdoor wireless system was developed using Wireless Sensor Networks (WSNs) equipped with low-cost sensors (PM_2.5_, CO, and NO_2_, relative humidity, and temperature). This system differs from other existing systems in that it addresses the needs of the Malaysian traffic police and is aimed at mobility and efficient data communication regarding air pollution. The system uses optical light scattering sensors for the PM monitor and electrochemical sensing principles for CO and NO_2_ monitors. Communication wise, a Wireless Sensor Network (WSN) is used with Arduino as its microcontroller and low-cost sensors for the air pollutants. This system has been used for a number of research projects and has been proven to work well for multiple purposes [21]. 

### 2.2. Importance for Traffic Police

Since air pollution is often coupled with congested traffic in densely populated areas, traffic police officers in Malaysia are overwhelmed by the consequences of working to regulate traffic congestion [14], as the diesel engines in freight and passenger vehicles produce extremely fine particles that are hazardous to human health [22]. The impact of ambient air pollution can be seen annually among traffic police officers [4,5]. However, it is difficult to limit or regulate personnel’s exposure to air pollutants as their work demands them to be outdoors for most of the time. Therefore, the alternative solution is to monitor their exposure and to have them act accordingly when the reading is high. To address this issue, this research offers a conceptual design of a wireless outdoor individual exposure indication system prototype for traffic cops based on their needs while on duty, as well as field testing of the prototype’s performance.

### 2.3. The Conceptual Design 

**Figure 1:**
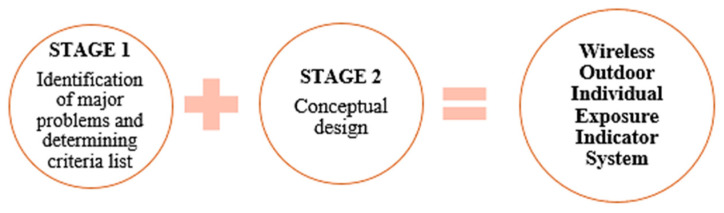
Summary of the design process.

The conceptual design of the individual outdoor exposure indicator using a wireless system involves several stages as summarized (Figure 1). Stage One is focused on the level of exposure of traffic police to outdoor air pollution in a heavy traffic area using secondary data from DOE, which is presented in another article by the same author [14]. Once the current situation of the traffic police is identified in Stage One, the next stage involves recognizing the occupational hazards faced by Malaysian traffic police in current situations using previous literature as Stage Two, which is discussed in a recent publication [23]. The review in Stage One and Stage Two presents a better knowledge of the actual situation and the working environment [24]. Both stages are critical for identifying and recognizing the Malaysian traffic police’s criteria and needs for the individual outdoor exposure indicator using a wireless system. Data from these studies were analyzed and used as an input for the third stage which is the conceptual design. A closed discussion was held a few times in January 2019 at Bukit Aman Headquarters; in which the research team (a representative from an Electronic System Design and Software Development company, a Supervisory Committee, and a representative of Royal Malaysian Police) successfully worked together to narrow down the criteria that were mutually agreed on, based on the scope and limitations of the systems developed in previous studies. The discussions were carried out in order to determine the most suitable and essential criteria including the prototype’s components. The explanation of the concept development is further discussed in detail in another recent publication by the same author [25].

**Figure 2:**
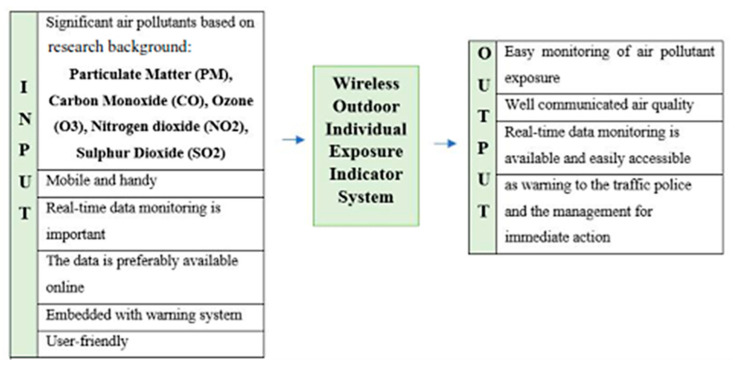
Criteria of the proposed system.

These two stages illustrate a list of criteria (Figure 2). These criteria are subsequently expanded into a Product Design Specification (PDS) document, which serves as a reference throughout the prototype development process. Thereafter, a wireless outdoor individual exposure indicator system prototype is developed, and initial testing to test its functionality is conducted. The development process is a continuous process starting with the PDS development to the field testing. The field-testing stage is critical for ensuring that the system is free of mishaps [26].

### 2.4. Experiment Location

The next stage is to conduct a use and field evaluation process which is undertaken according to the guidelines of the National Exposure Research Laboratory of the United States Environmental Protection Agency [16] and the United Kingdom of Environmental Agency Technical Guidance Note [27].

The prototype involved in this study was designed utilizing user-centered concepts with the goal of being a low-cost mobile monitoring device, and detailed designs have been published by the same author elsewhere. The study tested the accuracy of the prototype’s temperature, relative humidity, PM_2.5_, CO, and NO_2_ measurements by comparing them with measurements from DustTrak II for PM_2.5_ and data from DOE for the rest of the parameters. Table 1 shows the parameters and the instruments used for comparison.

Malaysian DOE is a department that is responsible for monitoring the air quality in Malaysia. Air quality is continuously and routinely monitored to detect any changes in the air quality status which may cause harm to human health and the environment [28]. This monitoring is known as Continuous Air Quality Monitoring (CAQM). The Department of Environment (DOE) tracks the ambient air quality of the country across a network of 51 stations. In order to identify any major change in air quality that could be detrimental to human health and the environment, these monitoring stations are strategically located in suburban, traffic, and industrial areas. Of the 51 stations built in Malaysia, 26% are industrial stations, 57% are residential, 2% are traffic, 2% are background stations, and 13% are PM_10_ stations. These are methods of high resolution which provide continuous records of the levels of pollutants. With minimal operator interference, they may work over prolonged periods (weeks or months) [29]. They have a high degree of accuracy of measurement and have levels of detection around one order of magnitude or more below normal levels of background. These are the most expensive methods of tracking, as would be expected. For good data quality, a high standard of maintenance, calibration, and operational and quality control procedures are required [30]. The system consists of a sensor mode gateway and back-end platform controlled by the lab view software system, in which the data can always be recorded in a database. The system is installed to the main road in the city to observe the carbon monoxide concentration caused by the vehicle, but most of these strategies are expensive, provide low resolution collected data, and these stations are less densely deployed [31].

The DustTrakTM II Aerosol Monitor 8530 which is manufactured by TSI Incorporated, Minnesota, United States of America (USA) is a data-logging, single-channel, light-scattering laser photometer that captures a gravimetric sample and provides real-time aerosol mass values. It makes use of a sheath air system to keep the optics clean for increased dependability and little maintenance. This system separates the aerosol in the optics chamber. It is appropriate for both sterile office settings and tough industrial locations, as well as for outdoor applications and construction and environmental sites. Dust, smoke, fumes, and mists are among the aerosol pollutants that the DustTrak II Aerosol Monitor monitors. With gravimetric sampling, the DustTrakTM II Aerosol Monitor provides real-time aerosol mass data for particle sizes ranging from 0.1 to 10 m [32]. 

**Figure 3:**
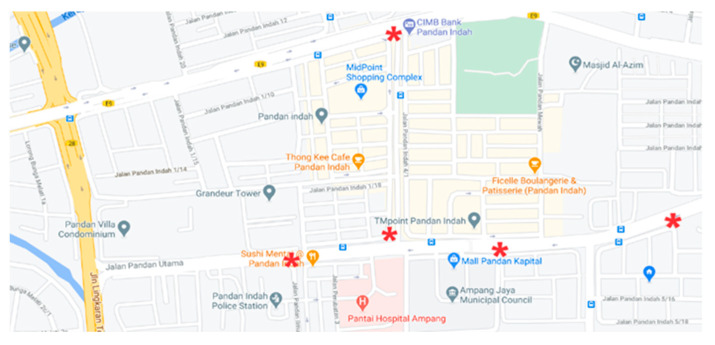
Stationary field-testing locations. The red star indicates the exact position where the prototype was deployed. (Source: Google Maps available at https://www.google.com/maps/place/Pandan+Indah,+Kuala+Lumpur,+Selangor/@3.1311177,101.7523725,16z/data=!4m5!3m4!1s0x31cc365f86dd4897:0xdb6d184b895e07c7!8m2!3d3.133892!4d101.7516751, accessed on 10 November 2020).

Due to the Movement Control Order (MCO) in Malaysia following the COVID-19 pandemic, a field research study is restricted; thus, the monitors were installed within a residential area. For the stationary testing, the monitors were set to measure pollutants simultaneously at 1 min intervals for eight hours daily (working hours), for 16 consecutive days (from 13 November 2020 to 28 November 2020). Figure 3 shows the location of the stationary testing for the collocation. The monitors were assembled at a junction in a high-density residential area, in a suburban town, Ampang Jaya, near the Kuala Lumpur city center. The monitors were located at an approximate height of 1 m. Care was taken to ensure the monitors were placed away from any direct pollutant sources, heat sources, and ventilation ducts or openings.

**Figure 4:**
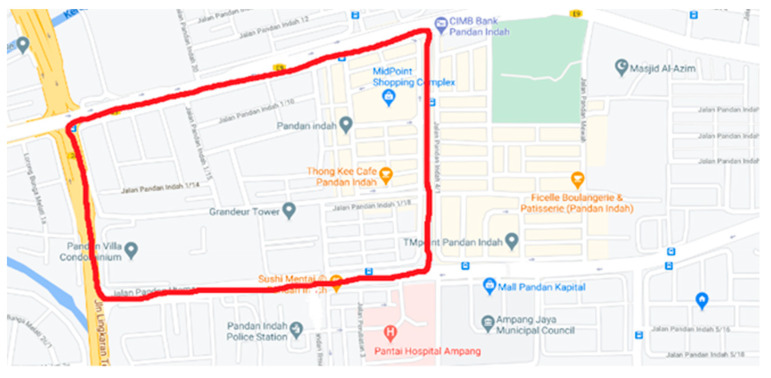
Routes followed in the mobile testing around the area of Pandan Indah, on 24 November 2020. (Source: Google Maps available at https://www.google.com/maps/place/Pandan+Indah,+Kuala+Lumpur,+Selangor/@3.1311177,101.7523725,16z/data=!4m5!3m4!1s0x31cc365f86dd4897:0xdb6d184b895e07c7!8m2!3d3.133892!4d101.7516751, accessed on 10 November 2020).

To test its functionality while mobile, a mobile monitoring was deployed around the same area due to the MCO. The mobile testing was carried out on 24 November 2020 for two continuous hours. The routes taken during mobile testing are shown in Figure 4.

### 2.5. Data Analysis

The data from both monitors were exported into IBM SPSS Statistics Version 24 for statistical analysis. The Kolmogorov Smirnov test rejected the hypothesis of normal distribution. Therefore, the Spearman’s rank correlation was carried out to determine the correlation of both monitors. The linearity among the monitors was tested using linear regression.

## 3. Results

### 3.1. Conceptual Design Based on PDS

**Figure 5:**
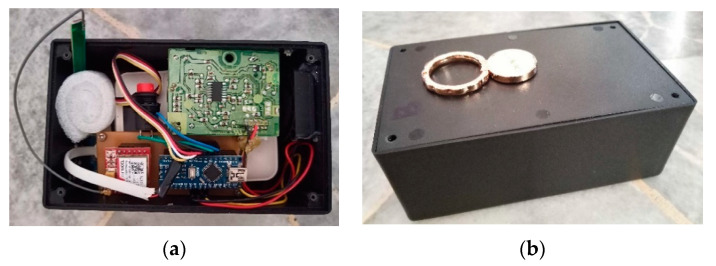
Prototype (**a**) Internal Connection. (**b**) External case.

A detailed product design specification (PDS) was developed based on the criteria acquired from previous research studies. PDS is used to analyze, design, manufacture, and construct a component to achieve a specified degree of efficiency, performance, or quality [33]. The PDS criteria were chosen for the development of the individual outdoor exposure indicator using a wireless system. The details are tabulated in Table 2. The assembly of the prototype of the individual outdoor exposure indicator using a wireless system to measure PM, CO, and NO_2_ levels and other factors of influence such as temperature and humidity among the Malaysian traffic police and the external view is illustrated (Figure 5). 

**Figure 6:**
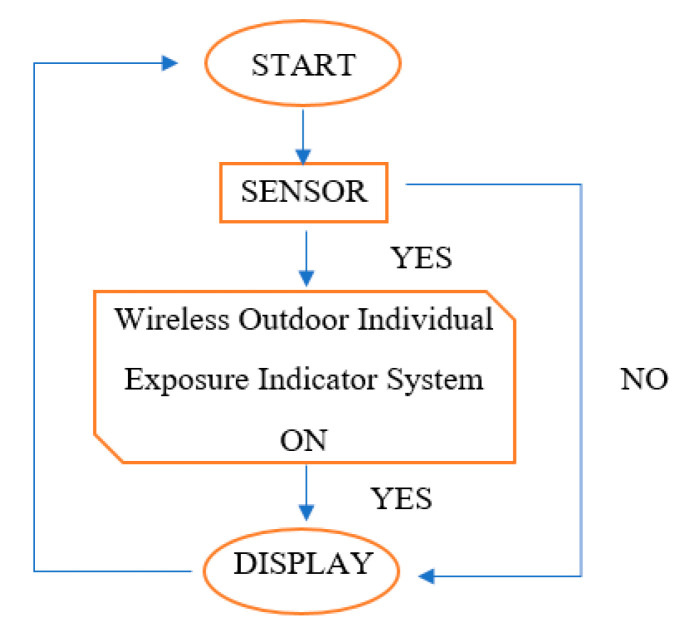
The flow of the system.

**Figure 7:**
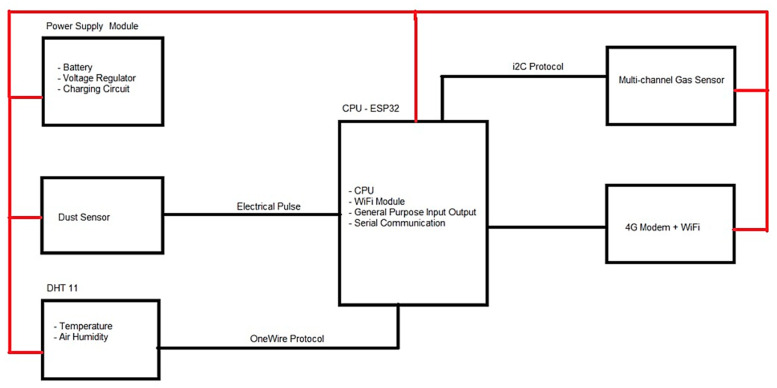
Internal Architecture of the Wireless Outdoor Individual Exposure Indicator System Prototype.

It is important to understand the requirements and functionality of an outdoor individual exposure indicator system to monitor the outdoor air quality. The system flow for monitoring outdoor air quality is demonstrated (Figure 6). The flow starts with the switching on of the device, and all the sensors will automatically run to measure the data which are displayed on the website. If there are no data displayed or captured on the website or apps, the user needs to restart the system by switching it off and on again. The design, as discussed, enables the delivery of real-time data and information that can be accessed from personal computers and smartphones [34]. This system uses minimal human interaction with the device, has a small dimension, and is lightweight as well as mobile. The complete internal architecture of the system design is illustrated (Figure 7).

**Figure 8:**
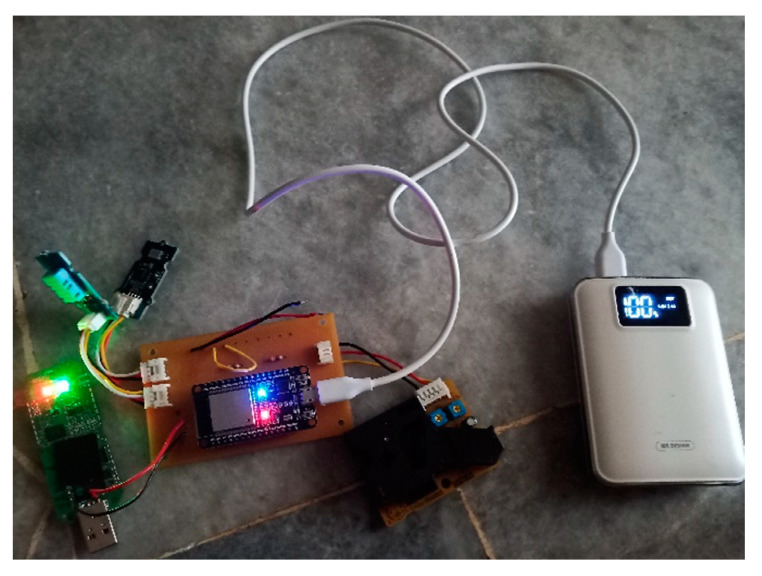
Connectivity of the individual outdoor exposure indicator using wireless system.

Specifically, the proposed system consists of sensor nodes equipped with a power supply, CPU, 4G Wi-Fi Modem, and three sensors—each measuring PM, NO_2_, and CO, temperature, and humidity. The connectivity of the prototype components to further understand its working principle is demonstrated (Figure 8). The sensors collect data in an analogue pattern, which are later converted into digital data by the CPU and then sent wirelessly to the web server by the 4G Wi-Fi Modem. Data from the three sensors will be displayed in the form of tables in Excel and PDF through the webserver. The data can also be accessed by smart devices, implying that it can be viewed anywhere in real-time and online. The functions of each sensor node were tabulated (Table 3).

According to Austin [43], within a set of predetermined settings, the Shinyei sensors can be dependably utilized to detect particles with sizes ranging from 0.5 to 2.5 µm. In addition, after translating each sensor response to a mass concentration using a linear regression, as mentioned in the techniques section, the accuracy of the mean response of these 20 sensors in the linear 0–50 µg/m^3^ range was estimated to be 9%. This is consistent with the EPA’s description of particles in the respirable range.

**Figure 9:**
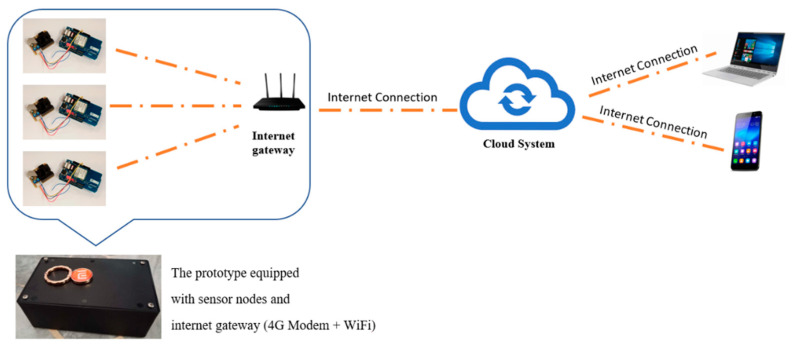
Cloud system used by the prototype.

The system is connected to the end user application (website and smartphone application) to display the measurement data from the sensors. First, the initial component (sensor network) comprises multiple nodes that integrate various types of sensors. The network nodes were connected using the Arduino processor and a gateway was required to receive data from each node and retransmit it to the cloud system. The gateway must also ensure that packets from network nodes and the cloud system are received. Both the sensors and the internet gateway were integrated within the prototype developed in this study. The cloud system is also in charge of receiving data from the sensor network and delivering particular data storage, categorization, and request services. Finally, end-user software applications that provide services for requesting data from the cloud system make up the final component. The prototype used a cloud system to provide a clearer understanding of how it works (Figure 9).

**Figure 10:**
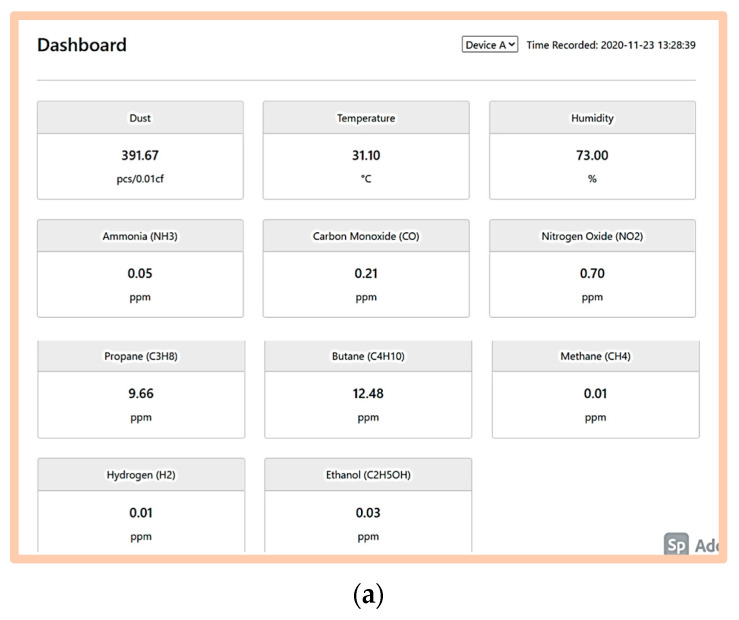

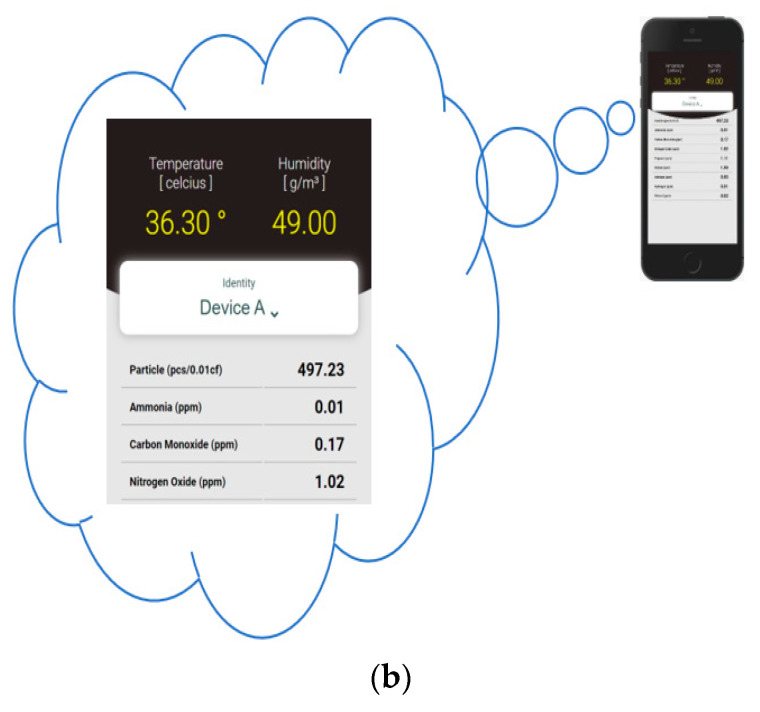
Wireless Outdoor Individual Exposure Indicator System display on different platforms. (**a**) Display on a website. (**b**) Display on smartphone application.

The prototype uses the Internet of Things (IoT) concept to transmit the data wirelessly by using the 4G Modem + Wi-Fi components, which transmit the data to other devices. The reading from the prototype was displayed in two manners: (i) the website and (ii) the smartphone application. The data measured are displayed on a website at https://airpolutionmonitor.web.app (accessed on 23 November 2020). The interface of the webpage for the air quality monitoring system and the display on a smartphone application are illustrated (Figure 10).

A warning system was also integrated, where the traffic police in the location with a high level of PM_2.5_ receive a warning in terms of messages to take actions, such as leaving the area and switching with other colleagues. Before the system is ready, it must be tested prior to launching it as a reliable measuring device. The developed prototype was evaluated for field testing to confirm that the system captures and transmits all the data to the software applications and is critical for ensuring the system is free of errors [25].

### 3.2. Measurement Results 

**Figure 11:**
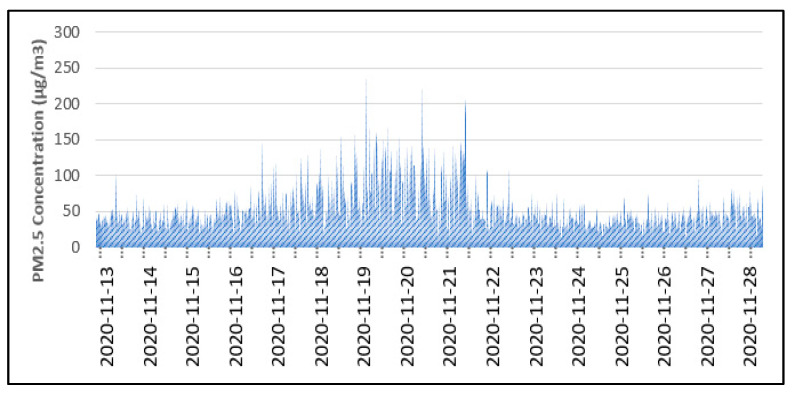
Temporal distribution of PM_2.5_ during the testing.

Figure 11 shows the temporal distributions of PM_2.5_ during the collocation campaign for 16 days from 13 November 2020 to 28 November 2020 (stationary field testing). The data displayed are the static data collected at a junction in a suburban town, Ampang Jaya, near the Kuala Lumpur city center. This shows that the prototype was able to serve its function of collecting PM_2.5_ data in an environmental setting similar to the working environment of the Malaysian traffic police. The trend indicates an increased concentration of PM_2.5_ on working days from 16 November to 20 November 2020, while fewer vehicles were seen on site in the following week due to stricter CMCO regulations and workers’ year-end holidays.

**Figure 12:**
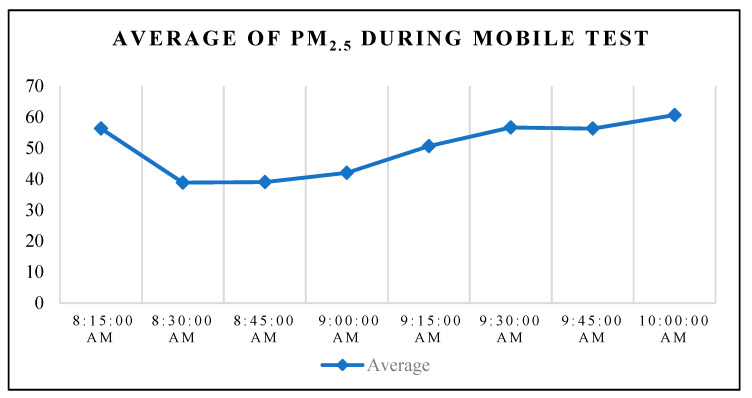
Hourly average of PM_2.5_ during the mobile test run.

The average readings of PM_2.5_ during the mobile test on 24 November 2020 can be seen in Figure 12. The trend shows that the prototype was able to capture data and transmit it to the website and smartphone applications which were accessible online. To further analyze the performance in terms of accuracy, the measurements from both the prototype and the DustTrak (reference instrument) were compared and are displayed in the graph below.

**Figure 13:**
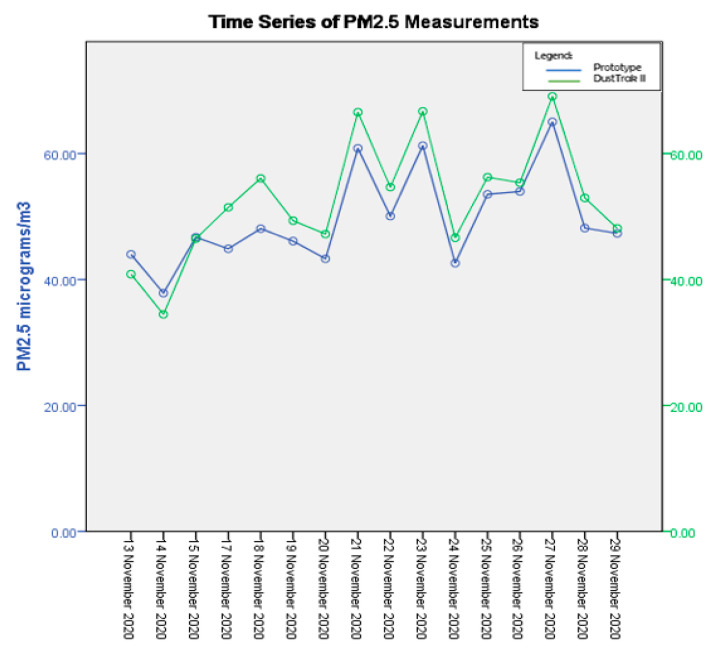
Daily average of PM_2.5_ mass concentration for the prototype and the DustTrak.

**Figure 14:**
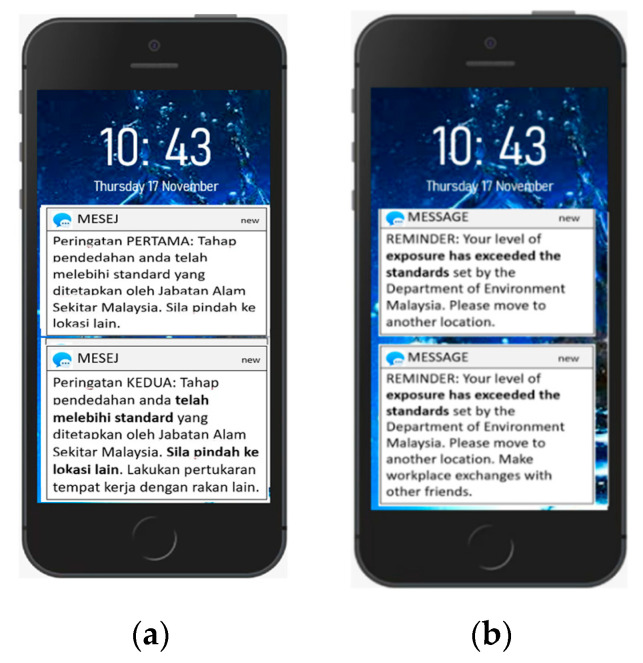
The smartphone display messages (**a**) in Malay language, and (**b**) in English Language.

Figure 13 shows the daily average PM_2.5_ mass concentration for the prototype and the DustTrak (reference instrument). Over the 16 days of the testing period, the PM_2.5_ daily limit of 35 µg/m^3^ was exceeded when compared to the Malaysian New Ambient Air Quality Standard 2020 [44]. This was due to the associated traffic emissions and meteorological conditions during testing; the result is consistent with that of a past study in 2016 [45]. Testing occurred at an average temperature of 34.4 degrees Celsius with an average relative humidity of 56.6%. The prototype is extra privileged which allows notification in both the website and smartphone application whenever the average of PM_2.5_ exceeds the daily limit of Malaysian New Ambient Air Quality Standard 2020. Figure 14 show the smartphone’s message display during the collocation. By having such system, it is possible to take immediate action, such as avoiding polluted areas or staying at home as agreed upon by Tomić et al. in their study on Supervisory Control and Data Acquisition system [22].

**Figure 15:**
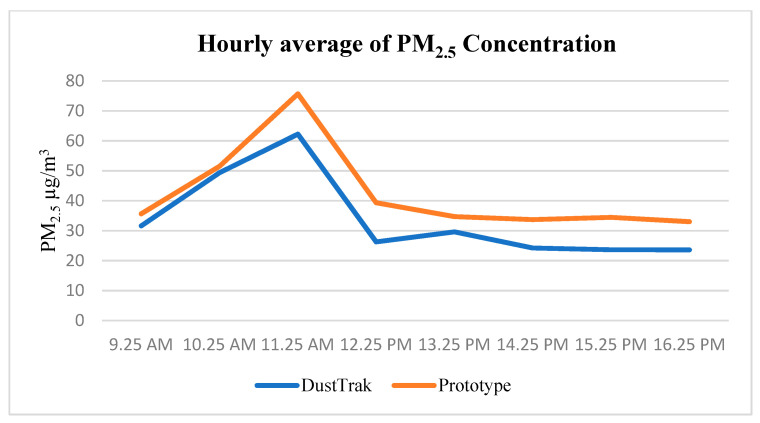
Hourly average of PM_2.5_ mass concentration.

Figure 15 shows the hourly average PM_2.5_ mass concentration for the DustTrak and the prototype. The range of reading for the prototype was recorded from 33.01 to 75.7 µg/m^3^, whereas the range for the DustTrak II was recorded from 23.61 to 62.24 µg/m^3^. The trend shows similar readings from all the instruments. The finding indicates reliable measurements from the prototype. The similarity was found with previous works of literature [46,47,48].

**Figure 16:**
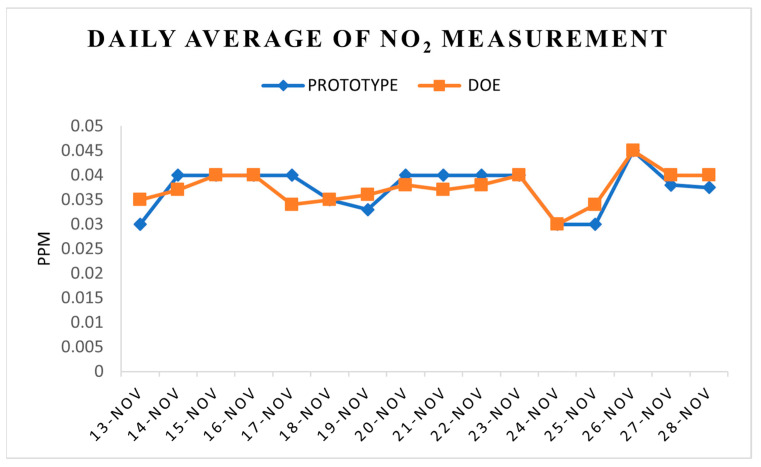
Daily average of NO_2_ mass concentration.

**Figure 17:**
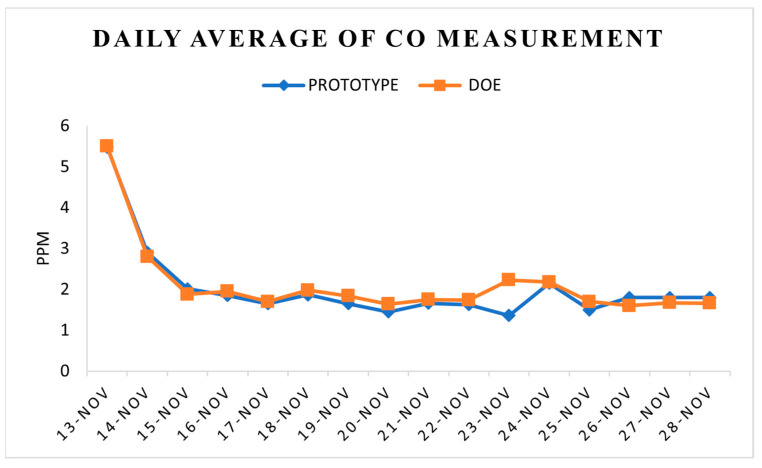
Daily average of CO mass concentration.

Hourly average NO_2_ and CO measurements in Figure 16 and Figure 17 were derived from the prototype itself and the DOE’s records. There was a similar range of readings for both the prototype and the DOE’s records, i.e., 0.030 to 0.045 ppm for NO_2_. As for CO, the range recorded by the prototype was from 1.36 to 5.4 ppm, whereas the range from 1.64 to 5.5 ppm was DOE’s records. The results suggest similar readings from all the instruments, which also indicates that the sensors used in the prototype are dependable. This agrees with the results reported by other researchers [49,50].

### 3.3. Correlation between the Prototype and the Reference Instrument for PM_2.5_, NO_2_, and CO

A Spearman’s rank-order correlation was run for further analysis to determine the relationship between the prototype and the DustTrak (reference instrument) for PM_2.5_ measurement. There was a moderate, positive correlation between the prototype’s data and the DustTrak’s data, which was statistically significant (rs = 0.207, *p* = 0.019), as shown in Table 4 below. This is consistent with similar conclusions made by other researchers [49,50,51,52].

For CO and NO_2_ measurements, Spearman’s correlation was carried out for correlation testing. There was a moderate, positive correlation between the prototype’s data and the DOE’s records, which was statistically significant for both NO_2_ (rs = 0.576, *p* = 0.02) and CO (rs = 0.545, *p* = 0.04), as shown in Table 4. Clearly, the sensors in the prototype provide reliable and accurate readings. The positive correlation is supported by similar studies using the same technology [46,47,48].

### 3.4. Linear Regression Test for Calibration

The field calibration was performed in September 2020, where the prototype was placed next to a reference instrument (DustTrak) in Ampang Jaya in a near-road location just east of Kuala Lumpur city center. The prototype was positioned at about the same height and faced the same orientation as the DustTrak. The horizontal gap between the prototype and DustTrak was approximately 30 cm. The calibration in the field was performed for 128 h.

Linear regressions were used to evaluate the calibration of prototype sensors; the independent variables were signals and data from prototype sensors, while the concentration of air pollution from DustTrak was the dependent variable (Table 5). The regression equation was

Hourly average for Prototypemodel1 = 9.188 + 0.768DustTrakvalue(1)

The *p* value < 0.001 rejects the null hypothesis.

**Figure 18:**
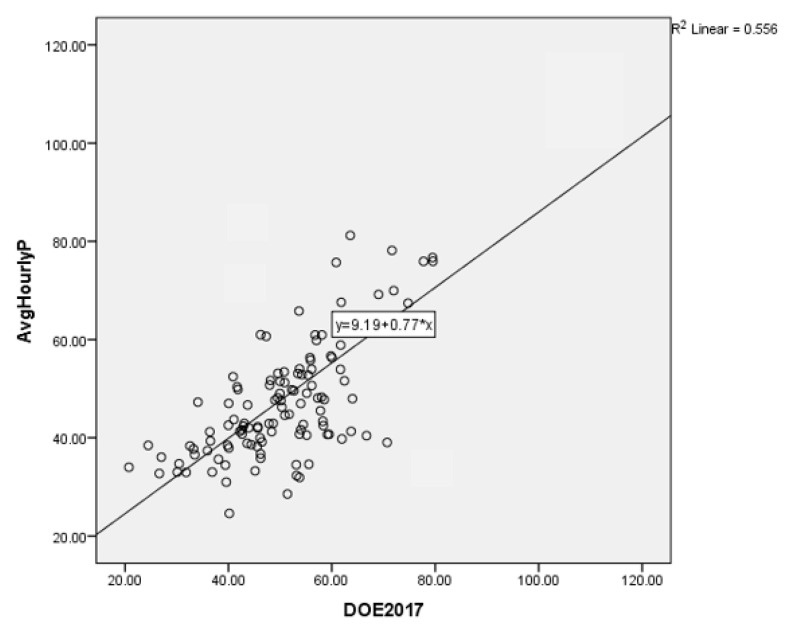
Regression of pollutant concentrations PM_2.5_ prototype compared to DustTrak reference monitors. The linear regression equation is issued.

The PM_2.5_ sensor had a varied performance with an R^2^ = 0.556. The PM_2.5_ increased from 0.542 to 0.593. As the *p*-value is greater than 0.05, NO_2_, CO, temperature, and relative humidity were not included in the model. The PM_2.5_ sensors presented a high R^2^ (>0.5), which indicates good agreement with the fitted model shown in Figure 18. Due to the COVID-19 pandemic and financial restrictions, the testing of the prototype in this study was conducted in a short period of time. Although the collocation is carried out over a period of 16 days, the outcomes are regarded as reliable. The research was conducted while Malaysia was under a MCO because of the COVID-19 outbreak at the time of the study, which also contributed to the research restrictions of time limits for data collection. It was highly suggested that the prototype should be tested for a longer period (more than 6 months) and that a more detailed analysis should be conducted to provide more evidence regarding the prototype performance.

Before conducting any measurements with these low-cost sensors, calibration in the field is highly recommended [46]. The model can be used as a calibration function as significant improvements were seen after using them as stated by Karagulian et al. [53]. Since the sensor’s sensitivity varies over time, which we estimate to be ~3 months, the calibration must be carried out regularly. We conclude that the prototype can achieve accurate ambient air quality measurements for most of the pollutants examined with adequate calibration and monitoring of sensor efficiency.

## 4. Conclusions

The development of a wireless outdoor individual exposure indicator system prototype allows for an inexpensive tool to capture real-time data and information that can be accessed online using computers and smartphones. As an alternative approach to overcoming the numerous problems faced by the Malaysian traffic police in tracking their exposure to air pollution, this device is extremely important as such technology is very limited in Malaysia. In this study, the prototype system was presented to address its field performance by collocation with reference monitors. It can be used as a portable monitoring unit in real world scenarios. For this study, the test was performed to simulate the Malaysian traffic police’s working environment, which involves roadsides with dense traffic. 

Although the system was evaluated in a relatively short period of time, and at limited locations due to the pandemic, this work is the first step in determining the system’s suitability for different field applications. In conclusion, the advantages of using the system to evaluate various pollutants have been proven. All the tested data significantly correlated with each other (PM_2.5_-rs = 0.207, *p* = 0.019; NO_2_-rs = 0.576, *p* = 0.02 and CO-rs = 0.545, *p* = 0.04). The calibration model was obtained from linear regression. The developed prototype demonstrates that an individual outdoor exposure indicator using a wireless system is suitable to ensure a safe and healthy working environment for the Malaysian traffic police. The system is able to measure PM_2.5_, NO_2_, and CO data with sufficient accuracy, so it is a reliable tool for monitoring the exposure to air pollution, which will benefit traffic police officers on duty in Malaysia. Nonetheless, for comprehensive monitoring, it is advised that several important sensors such as sulfur dioxide and carbon dioxide be included in the system. It is worth noting that adding more sensors will elevate the system’s cost, which is a critical criterion for the prototype. This study also has limitations because only a small part of Malaysia—not the entire nation—was examined. Due to the COVID-19 pandemic at the time of the study, Malaysia was under an MCO, which also contributed to the study’s weakness: inadequate equipment and time constraints for data collection. As a result, several innovative and trustworthy solutions were created to satisfy the study’s objectives. The prototype is intended to be an engineering intervention that will assist the Malaysian traffic police in tracking their workplace outdoor air pollution exposure. There is a suggestion for advancement to include all the air quality indicators, such as sulfur dioxide, ozone, and carbon dioxide. Keep in mind that adding all the sensors may result in an increase in cost; therefore, a thorough assessment of the PDS is suggested for future research. A cost rise must be avoided because the prototype concept is a low-cost gadget. In addition, a longer testing period (more than 6 months) for in-depth testing is required to demonstrate the long-term sensor performance and the sensor reliability, which is not possible for the present study due to the pandemic and MCO in Malaysia. To enhance the prototype of the individual outdoor exposure indicator using a wireless system, which is easier to use and more durable, the design must be improved. One possible enhancement is to incorporate a wearable concept. Finally, future studies are recommended to validate additional assessments by using the calibration model. Such data can be used to ensure that the relevant laws and guidelines protect the safety and health of traffic police officers.

## Figures and Tables

**Table 1:** Parameters and reference monitors used in the study.

Parameters	Reference Monitor
Particulate Matter with diameter of less than 2.5 micrometers (PM_2.5_)	TSI DUSTTRAK II Aerosol Monitor 8532
Carbon Monoxide (CO)	Data provided by Malaysia’s Department of Environment (DOE)
Nitrogen Dioxides (NO_2_)	

**Table 2:** PDS for the individual outdoor exposure indicator using wireless system.

	Criteria	Requirements
1	Performance	Using a cellular connectivity and rechargeable power supply; Data to be measured and uploaded at 5 s time interval.
2	Environment	It can withstand relatively high and low temperatures (0–60°), vibration, and shock.
3	Life in service	It can withstand up to 2 to 5 years with regular calibration as claimed by the manufacturer, SHINYEI Technology Co., Ltd. (Osaka, Japan). Further long-term experimentation needs to be conducted to support the claim.
4	Maintenance	Easy regular calibration is required for accuracy of data and efficient performance.
5	Standard & Specification	Able to measure important parameters (according to the Malaysian Department of Environment and US EPA) of air pollution: Particulate Matter (PM_2.5_) Carbon Monoxide Nitrogen Dioxide Temperature and humidity Multi gases (Propane, Butane, Ammonia)
6	Size	Handheld size and mobile
7	Weight	Lightweight material (<1 kg)
8	Target Product Cost	Much lower cost compared to the conventional air monitoring station.
9	Materials	It must be made from a strong, lightweight, water-resistant, and shockproof housing for all the sensors to be embedded inside.
10	Customer	Traffic policemen (exposed to outdoor air pollution).
11	Installation	Easily operated with a switch on the casing and is powered using a power bank. Once switched on, the data are uploaded to the website and mobile application. The website serves as a platform to collect and store the measured data at 5 s intervals. The device is wearable and mobile, does not need to be fixed to anything.

**Table 3:** Functions of sensor nodes.

Items	Functions
Power Supply Module Battery	A battery is a device that stores chemical energy and converts it into electrical energy which produces an electrical current that can be used to do work [35].
Voltage regulator	The voltage regulator produces an output voltage of a fixed magnitude that remains constant in relation to changes in its input voltage or load conditions [36].
Charging circuit	Functions switch between the conductive state to enable the current flow and the non-conductive state to prevent the current flow [37].
Dust Sensor (Shinyei PPD42NS Low-Cost Particulate Matter Sensor)	Capable of detecting dust in the environment using an optical sensing method. A photosensor and an infrared light-emitting diode, known as the IR LED, are optically arranged in the dust sensor module. The photosensor (PT) detects the reflected IR LED rays which are bounced off of the dust particles in the air [38].
DHT 11 (Temperature and air humidity)	A digital temperature and humidity sensor is used to measure the surrounding air and emits a digital signal to the data pin (no analog input pins are needed) [39].
CPU ESP32 Wi-Fi Module	It can either host an application or import all WiFi networking functions from another application processor [40].
General Purpose Input Output	Sends signals from sensors to the system [41].
Serial Communication	To transmit or receive one bit of data at a time [24].
Multi-channel Gas Sensor	A sensor for the environment which is capable of detecting multiple gas types. Three gases can be measured simultaneously due to its multi-channel; hence, it is able to monitor more than one gas concentration [42].
4G Modem + Wi-Fi	Transparent communication between mobile phones and serial devices [24].

**Table 4:** Correlation test for PM_2.5_, NO_2_, and CO.

Variables	Frequency	r Value	*p* Value
PM_2.5_ Prototype/DustTrak	128	0.216	0.014 *
NO_2_ Prototype/DOE	128	0.576	0.02 *
CO Prototype/DOE	128	0.545	0.036 *

* Correlation is significant at the 0.05 level (2-tailed).

**Table 5:** Linear regression.

Variables	b (95% CI)	*t* Statistics	*p* Value	r^2^
DustTrak	0.768 (0.684, 0.889)	12.552 *	<0.001	0.556

* Simple linear regression.

## Data Availability

Data sharing is not applicable to this article.
